# Virtual vs. standardized patients - a preliminary comparison of advantages and disadvantages of a VR and a conventional learning unit for medical students in child and adolescent psychiatry

**DOI:** 10.1186/s12909-026-08760-4

**Published:** 2026-02-17

**Authors:** Linda Graf, Vera Golz, Sophia Prehn, Maic Masuch, Gertraud Gradl-Dietsch

**Affiliations:** 1 Entertainment Computing Group, University of Duisburg-Essen, Forsthausweg 2, Duisburg, 47057 Germany; 2 Department of Child and Adolescent Psychiatry, University of Duisburg-Essen, Wickenburgstraße 21, Essen, 45147 Germany

**Keywords:** Teaching with technology, Educational intervention, Virtual patients, Emotional virtual agents, Virtual reality

## Abstract

**Background:**

Constructivist learning theories are getting more and more attention in medical education, as generating understanding and knowledge through personal experience seems to be promising. In child and adolescent psychiatry, simulation-based training is challenging, as students’ access to real young patients is limited and there are no standardized patients of that age. Hence, we see Virtual Reality (VR) presenting virtual patients as a means to address these issues. VR enables the standardization of personal encounters in an adaptive learning environment that is cost-effective, scalable and applicable in a standardized way for different learners. Even though virtual patients have become more and more popular in medical education, less studies investigated the strengths and weaknesses of those compared to conventional standardized patients.

**Methods:**

We present a within-subjects study with 50 students and compare our virtual patient system, a VR application that allows student to engage in a conversation with a virtual patient, with a conventional standardized patient unit. Students participated in small groups of up to five students alternately in the two units and filled out questionnaires regarding the estimated learning success, believability, empathy, and motivation subsequently. One student at a time conducted the interview with the virtual or standardized patient, while the others took on an observational role. Finally, all students were interviewed comparing both experiences.

**Results:**

Results showed that the standardized patients were rated significantly higher in all dependent variables. Qualitative results highlight an inappropriate speech recognition system as main reason why the virtual patient system proved to be inferior. Furthermore, we identified aspects that demonstrate advantages as well as disadvantages for both units each.

**Conclusions:**

In its current technical implementation, the virtual patient system could not match standardized patients in interactional and affective dimensions. Nonetheless, we identified several aspects, that could be beneficial over or supplementary to the use of standardized patients. Furthermore, we derived implications for the future design of virtual patient systems and in which situation they should be considered.

**Supplementary Information:**

The online version contains supplementary material available at 10.1186/s12909-026-08760-4.

## Background

Simulation-based teaching brings constructivist learning theory to the fore, which is based on the fact that understanding and knowledge are generated through personal experience [1]. Simulation-based learning in the sense of experiential learning strengthens the motivation of the learner and allows for critical examination as well as the development of new concepts and hypotheses [2]. Students who have never been confronted with patients may feel insecure in dealing with patients. Through standardized patients, learners can repeat practical exercises in a safe environment, test new hypotheses and initiate self-reflective processes. Communication and interaction are an integral part of all courses in child and adolescent psychiatry. Practical teaching poses a major challenge, as it requires the use of real or standardized patients (SP), since medical communication cannot be taught only through theoretical content. Depending on the pathology, real patients can only be interviewed by students to a limited extent. The organizational effort, including obtaining parental consent, is also comparatively high. There are practically no corresponding standardized patients for children and adolescents up to the age of 16, so young adults must assume this role instead, which is an approximation at best. During the pandemic, but also afterwards, the care of psychiatry patients has also changed due to the increased use of internet-based consultations, and students’ access was also completely limited.

Using Virtual Reality (VR) represents an innovative further development in teaching that addresses the aforementioned challenges and at the same time reflects simulation-based learning and technical progress in clinical care [3–5]. It enables a variety of different simulated encounters with virtual patients. Those are virtual characters, also known as agents, which are computer-controlled beings that can take, for example, the form of a virtual person or even a patient. Studies show that people treat virtual characters equally and perceive them as social entities if they are designed believable [6]. Believability can be increased, in particular, through the representation of different affective communication channels [7], which can also lead to virtual characters being able to trigger empathy in their counterparts [8]. Due to the high degree of sensory immersion and natural interaction, VR enables the simulation of personal encounters in an adaptive learning environment that is cost-effective, scalable and applicable in a standardized way for different learners [9].

In medical science, there are already some studies presenting virtual patient systems, including those with virtual patients as chatbots [10], or with virtual agents [11, 12], using computers [11, 12] or even VR headsets [3, 4, 13]. Over the past few years, multiple review articles have examined the effectiveness of virtual patient systems [14–19]. A systematic review conducted by Cook et al. [14] assessed virtual patient systems, focusing particularly on their effectiveness (learning outcome) in educating health professionals. Their findings indicate that while virtual patient systems demonstrate positive learning effects on clinical reasoning and knowledge acquisition when compared to no intervention, these effects are relatively minor in comparison to those of non-computerized interventions. Furthermore, many of the studies present their virtual patient system, but few compare them on a large scale with conventional methods such as standardized patients. For example, Han et al. (2021) [3] compared the use of standardized patients with a group of students who used a VR application in addition to a standardized patient to practice neurological examinations. The virtual patient was able to perform parts of the examination that the SP cannot simulate but can only describe (e.g., facial paralysis). The two conditions did not differ in terms of realness and satisfaction, but did differ in terms of the learning outcome (exam score), namely the students who also used VR performed better in the exam. Jiang et al. (2024) [20] point out that previous review studies examined the comparison of virtual simulations (including virtual patients) with traditional teaching methods, for example, reading case studies in class. These showed that virtual simulation reached at least comparable results regarding enhancing communication skills or knowledge. Moreover, they conducted a more specific comparison with mannequins and real persons (i.e., standardized patients). Their results showed no significant differences regarding any clinical competencies, but for communication skills they found an increased potential with evolving technologies. Also they conclude that virtual simulations have great potential regarding cost-effectiveness, as well as flexibility regarding time or space.

In summary, the evaluation of virtual patient systems is promising and can, therefore, also be of interest for the above-mentioned challenges in the field of child and adolescent psychiatry. First steps have already been taken by Mavrogiorgou et al. [5] with their virtual patient system in the field of teaching students in adult psychiatry, and Graf et al. [4] in the field of child and adolescent psychiatry. The latter presented initial design proposals for believable conversations with young virtual psychiatric patients. Their results showed that the virtual patient was perceived as highly believable by the students. The dialog system evaluation was positive overall – the conversations seemed satisfactory, but there was room for improvement in the flow of conversation, as the transitions and response times were not yet optimal. The design, therefore, showed great potential for a realistic and educational VR interaction for psychiatric training. But an effectiveness evaluation with regard to the students’ clinical skills, for example, within a comparison with conventional standardized patients is still pending.

Due to the current growth of Artificial Intelligence (AI)-supported systems, there are also initial studies in the field of virtual patient systems, for example, investigating the generation of natural responses from virtual patients using large language models (LLMs). Galland et al. [21] found that generated responses were rated more natural and coherent than real patient data. Dialog-act-conditioned models improved coherence, while type-conditioned ones enhanced naturalness, confirming that LLMs can believably simulate patient behavior when appropriately guided. Ayers et al. [22] investigated the difference between ChatGPT-based chatbot responses and responses from doctors in terms of the quality of information and empathy. The chatbot responses were preferred in 78.6% of cases, even though they were significantly longer, and were rated significantly higher than the responses from doctors in terms of both quality and empathy.

### Contribution

In this paper, we present a preliminary comparison of a virtual patient system (VR unit) with a standardized patient (conventional unit) to compare strengths and weaknesses of both approaches and derive direct recommendations for the further development of such virtual patient systems. In total, 50 medical students participated in a within-subjects design study experiencing both learning units in a randomized order. The primary educational aim of the learning units is the development of clinical communication skills, which are central to effective patient interviews. Although communication quality was not measured directly in this study, participants evaluated both units regarding their estimated learning success, motivation, believability of, and empathy towards the respective patients (virtual vs. standardized). All those variables are considered prerequisites for communicative competence in clinical interactions as they are reflect an active, emotional engaging, and self-directed learning process, which is central in constructivist learning. In this sense, our measures capture learners’ subjective experience of the interaction, which can support the development of communication skills even in a simulated context.

Hence, we ask the following research question: “*How does the use of a VR learning unit differ from a conventional learning unit?”* We formulated the following hypotheses:

1. * The VR learning unit differ from a conventional learning unit regarding estimated learning success & motivation.*
2.  *The VR learning unit differ from a conventional learning unit regarding believability and empathy transfer.*

Our results show that the virtual patient received lower ratings regarding all dependent variables compared to the standardized patients. However, an inappropriate speech recognition may have affect the assessment of the virtual patient. We also identified advantages of the virtual patient system, for example, a low entry barrier, a clear structure and standardization, as well as the believability of a young appearance, providing a suitable preparatory tool before engaging with standardized patients.

## Methods

To evaluate our approach of using VR and virtual patients, we conducted a study and compared our VR learning unit with a conventional unit, using standardized patients. Thereby, we investigated student’s estimated learning success, believability of and empathy towards the respective patients, as well as students’ motivation during the units. Therefore, we used a mixed-method approach combining quantitative (questionnaires), qualitative (interviews) and objective measurements (interaction logs).

### Participants

In total, 50 medical students (33 female, 12 male, 4 not specified) aged 22–32 (*M* = 25.4, *SD* = 2.80) participated in our study. The study was conducted during a seminar as part of the psychiatric curriculum with students in their 5th year. Students had little experience with child and adolescents psychiatry due to their semester, medium experience with standardized patients (*M* = 3.60, *SD* = 0.89), and rather low previous experience with VR (*M* = 2.42, *SD* = 0.77) measured on a scale from 1 (= no experience at all) to 5 (= extensive experience). During the three study days the 50 students were divided into 18 groups (three VR and three conventional units each day). Therefore, 9 students conducted the conversations with the virtual or standardized patients (*speaker*), and 41 students observed the conversations (*observer*).

### Learning units (VR vs. conventional)

For the practical learning units, we created a catalog of questions according to the AMDP system, that students should follow in the practical learning unit. For a more detailed description of the catalog of questions, see [4]. Following this catalog of questions, students could engage in a conversation either with a virtual patient (VR Unit) or with a standardized patient (conventional unit).

Therefore, we designed three patient personas: Caro (14 years) and Emily (10 years) both with depression, and Hannes (11 years) with social phobia (Fig. 2). Each of them presents unique challenges when it comes to conducting diagnostic conversations. While Caro shows a lot of the typical symptoms of depression, she is estimated to be diagnosed easier, but it is challenging to talk to her because of her suicidal tendencies. Emily displays only the minimal set of symptoms necessary for a depression diagnosis (according to ICD-10), making the diagnosis potentially more challenging. She is also reluctant and does not want to talk about her symptoms in much detail, what might make it even more difficult to talk to her. The difficulty in the conversation with Hannes is that he is shy and withdrawn due to his social phobia. For each patient we have compiled a catalog of symptoms, as well as a list of personal characteristics and information on family circumstances and hobbies.

We then gave these documents to the standardized patient actors for preparation and used them simultaneously as the basis for creating the response script for the virtual patient version of the personas.

#### Virtual patient - VR unit

The VR application runs on the Meta Quest 3. Students can interact using natural language input and, therefore, ask the virtual patient questions. The virtual patients are humanoid characters in a comic-style design, with particular attention paid to making them look youthful or childlike (Fig. 2). They are animated and synchronized with a human voice. They can, therefore, respond to a pre-arranged list of questions with pre-recorded answers. The AI tool wit.ai^1^ is used to recognize the questions and select the appropriate answers. A tablet can display hints for possible questions as well as entire questions for reading the questionnaire. For a more extensive description of the VR application, see [4].

#### Standardized patient - conventional unit

Standardized patients, meaning actors playing the role of the patient, able to simulate a conversation between a prospective physician and a patient, represent a conventional learning unit in medical education [3, 23]. In total, we booked six female and three male actors from our university’s standardized patient program for the three study days to represent our personas Caro, Emily, and Hannes. All of them got the patients’ script in advance and were prepared for the conversations at the study days in a one-hour session with a therapist.

### Assessment of the learning units

**Fig. 1:**
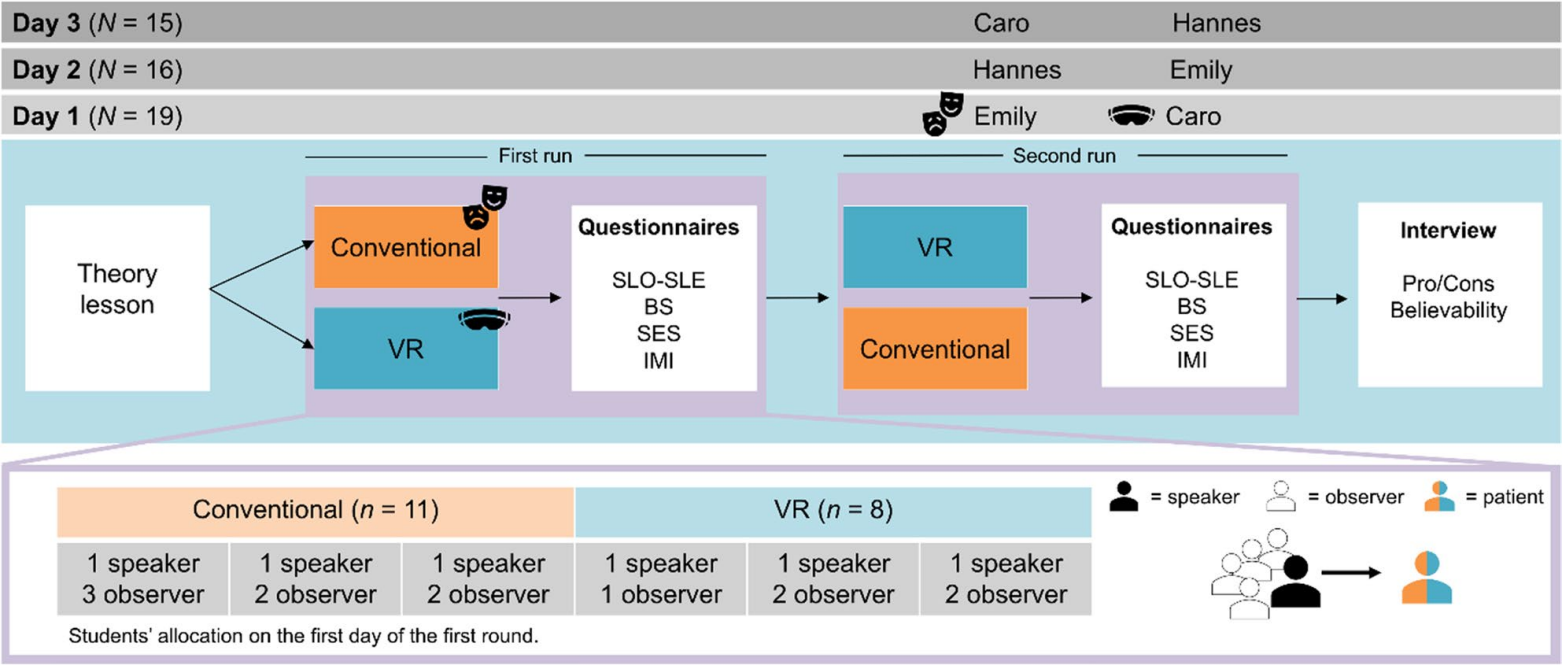
Illustration of the study procedure. For example, on study day 1, we had 19 students in total, from that 11 started with Emily in the conventional unit, and 8 students that started with Caro in the VR unit in the first run and then swaped

On each of the three study days, a different cohort of students took part in the course. The course started with a one-hour theoretical introduction lesson about “conducting difficult conversations in child and adolescent psychiatry”. After that, the course included a standardized unit and a VR unit in which the students had to apply the knowledge they had acquired from the theoretical lesson. On each of these three days, the students were divided into six small groups, three of which started with the standardized condition and three with the VR condition. Then, there was a cross-over. Accordingly, the study design follows a within-subjects design, as all participants went through both conditions in a randomized order to minimize potential learning and carryover effects. Thus, one half started with one condition and the other half with the other. We chose the within-design so that all students could experience both learning units and thus compare them directly with each other. Since the study was conducted within the course, all students should be given the same opportunities to gain experience. Within the small groups, one of the students was randomly assigned the role of the conversation leader (referred to as *speaker* ), while the other up to four students took on an observing role (referred to as *observers*). In order to ensure the comparability of the two learning units, the *speaker* remained the same in both conditions, so that one student conducted both interviews – with the standardized and the virtual patient. Due to the classroom-based implementation of the study, the number of students acting as *speaker* is substantially smaller than the number of *observers*. While this distribution reflects the practical constraints of the teaching setting and therefore was, it resulted in unequal group sizes across conditions. To ensure that both interviews were not conducted with the same patient persona, we had two of the three personas presented at each appointment (Fig. 1).

**Fig. 2:**
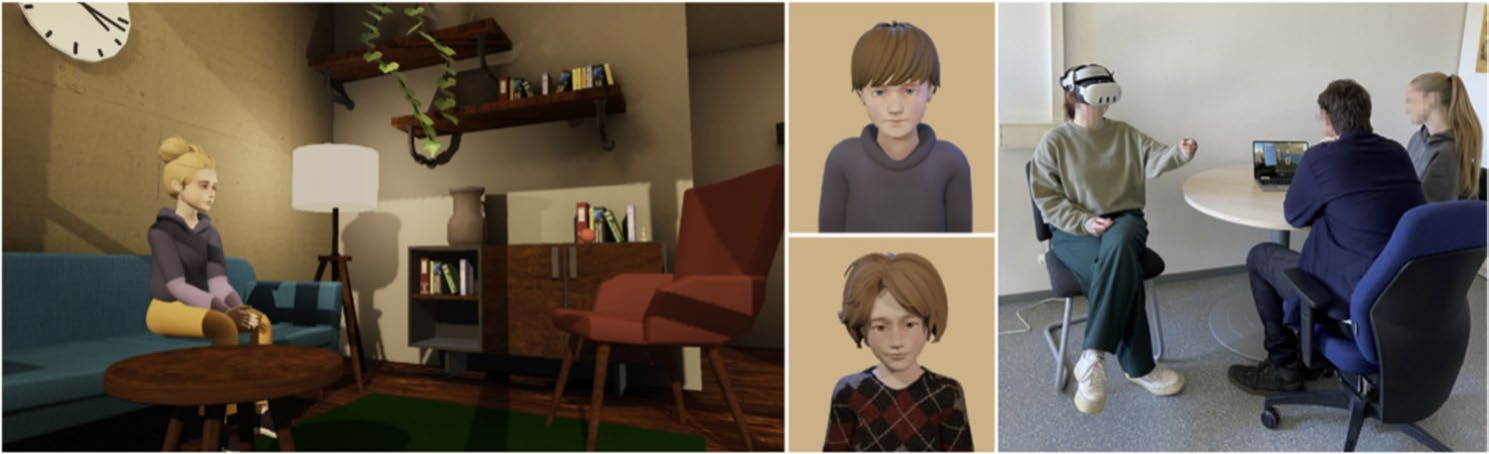
Left: Virtual patient ”Caro” in the virtual physician’s room; Middle: Virtual patients ”Hannes” (top) and ”Emily” (bottom); Right: Group of students in the VR group. (The photo was recreated with members of our team for the purpose of publication and, therefore, shows none of the participating students.)

After the theory lesson, the students split up into the six rooms (three times standardized, three times VR). They were greeted by an experimenter and gave their written consent for their data to be used for evaluation purposes in accordance with the latest version of the Declaration of Helsinki [24]. Students who did not agree to this took part in the two units without subsequently completing the corresponding questionnaires. The *speaker* was seated on a single chair and signed an additional consent form to be recorded during the interviews. The *observers* were given an observation sheet to fill out during the interview. This included noting symptoms that the patient either expressed verbally or showed non-verbally. The experimenter then briefly introduced the patient who would be the focus of the upcoming learning unit and who was scheduled to attend an initial consultation. They also provided information about the patient’s medical history as well as their personal and family history and previous treatments. The actor was then brought into the room in the standardized unit and the headset was put on in the VR unit. The *speaker* was equipped with a tablet, either real (standardized condition) or virtual (VR condition), that displayed the custom-designed questionnaire. The *speaker* was allowed – but not obliged – to use it as a guide during the session. While in the standardized condition the experimenter explained to the *speakers* how to navigate through the set of questions on the tablet, in the VR condition they received an introduction to the virtual tablet directly via a tutorial scene in VR. The conversations then began, which were interrupted by the experimenter only in the event of ambiguities or technical problems. In the VR condition, the *observers* were able to follow the conversation via a stream on a PC monitor (Fig. 2). Each conversation lasted approx. 20 min, after which the *speaker* had to click through a diagnosis catalog on the tablet based on the ICD-10 to make a diagnosis. The *observers* made their diagnosis on paper (without the help of ICD-10).

Then, all students (*speakers* and *observers*) filled out a questionnaire regarding their estimated learning success, believability and empathy towards the respective patient (virtual or standardized), as well as their motivation during the respective learning units. The second run then began, with the students switching from the three standardized groups to the VR groups and vice versa. They then completed the same questionnaires again as well as questions regarding demographics. Finally, the experimenter conducted interviews with the *speakers* and *observers* in each of the six groups to compare the two different learning units in terms of their advantages and disadvantages.

### Data collection

We used a quantitative survey of questionnaires and qualitative measures in form of a final interview to compare both learning units. Thereby, we used validated questionnaires [25–28], as well as self-formulated items and interview questions (see supplementary material). Furthermore, we analyzed log files of the conversations in VR to understand the conversations and identify errors such as unrecognized questions or incorrectly assigned answers.

#### Estimated learning success

To measure the estimated learning success, we used the subscale *perceived learning* of the Student Learning and Satisfaction in Online Learning Environments (SLS-OLE) instrument [25] rated on a scale from 1 (= do not agree at all) to 6 (= fully agree). Therefore, we replaced the word ’course’ with the corresponding learning unit, for example: ”*I am pleased with what I learned in the [VR/conventional unit]*”. Furthermore, we assessed the diagnosis students made after the interview with the patients to examine whether they correctly interpreted the standardized and virtual patients’ actions and characteristics.

#### Motivation

To measure the student’s motivation during the learning units, we used the subscale *interest/enjoyment* of the Intrinsic Motivation Inventory (IMI) [26] rated on a scale from 1 (= not true at all) to 7 (= agrees completely). The term ’activity’ was changed to the respective learning unit: ”*The [VR/conventional unit] was fun to do*”.

#### Believability

The believability of both patients (virtual and standardized) was measured with the Believability Scale (BS) [27] that was initially designed to measure believability of virtual agents. Here, we used the three subscales *behavior*, *emotion* and *overall* and adapted the items by replacing the term ’virtual agent’ with the respective patient (virtual or standardized). For example, ”*I felt the [virtual/standardized patient]’s behavior was appropriate to the context*” or ”*I felt that the [virtual/standardized patient] behaved like a real [patient]*”. All items are rated on a scale from 1 (= do not agree at all) to 7 (= fully agree). We chose the same scale across both conditions, to enable a direct comparison between virtual and standardized patients. As no established instruments are available that have been validated for both interaction modalities, scales originally developed for virtual agents were selected and linguistically adapted for standardized patients, as this required minimal conceptual modification compared to the reverse approach.

#### Empathy

To measure situational empathy for the patients, we chose the State Empathy Scale (SES) [28] and used the subscales measuring *affective* and *cognitive* empathy, both rated on a scale from 1 (= do not agree at all) to 5 (= fully agree). Again, we changed the term ’character’ to the respective patient (virtual or standardized): ”*I can feel the [virtual/standardized patient]’s emotions*” (affective) or ”*I can understand what the [virtual/standardized patient] was going through*” (cognitive).

#### Final interview

Subsequently, we conducted a semi-structured 10-minute interview with both speakers and observers comparing the two different learning units. Thereby, we used the following three main questions:

1. *Which learning unit (VR/conventional) did you like better and why?*
2. *How believable did the virtual character/actor portray the respective patient?*
  - *What did you particularly like?*
  - *What was missing?*

### Analysis rationale

For the analysis of the quantitative data, we conducted a mixed ANOVA with learning unit (VR vs. conventional) as a within-subject factor and participant role (*speaker* vs. *observer*) as a between-subject factor. The unequal distribution of speakers and observers represents a limitation of the present study. Although this imbalance was unavoidable in a realistic classroom scenario, it may have limited the statistical power for role-specific effects and interactions, potentially increasing the risk of Type II errors. The dependent variables were the *estimated learning success*,* believability*,* empathy*, and *motivation*. We tested for normality using the Kolmogorov-Smirnov test. For visualization, we present violin plots divided into speaker and observer for each learning unit. For the qualitative data, we transcribed the interviews and anonymized them with the help of the privacy-compliant automatic speech recognition software f4^2^ and assessed them with MAXQDA^3^. Three of the authors went independently through the transcripts and listed the reasons for the students’ preferences, benefits and downsides, as well as aspects regarding believability for the respective learning unit. Coding differences were subsequently discussed in joint meetings, and interpretations were refined iteratively until a shared consensus was achieved. In cases where initial agreement could not be reached, the issue was revisited in the context of the original transcript until a consensus was achieved.

#### Reflexivity statement

As this work involves a collaboration of scientists from different disciplines, the background of the different disciplines must be taken into account as they can potentially influence the interpretation of the results [29, 30]. For reasons of transparency, we would, therefore, like to briefly explain the disciplinary background of the scientists involved in this work. Four have a background in cognitive science and HCI, researching VR and virtual characters’ effects on users’ emotions, which informed the choice of a virtual patient–based study design and the selection of the respective measurement instruments for virtual interactions. One is a physician in the field of child and adolescent psychiatry, whose clinical expertise contributed to the design of realistic patient scenarios and ensured the clinical relevance of the learning tasks. During the interpretation of the results, researchers with the HCI background may have placed greater emphasis on agent-related interaction characteristics, whereas the physician may have interpreted the findings more strongly in terms of their implications for medical education and clinical training.

## Results

In the following, we present quantitative (questionnaires), qualitative (interviews), as well as objective (log data) results of our evaluation comparing the two learning units regarding the four overarching relevant constructs: *estimated learning success*, *motivation*, *believability* (of the respective patient), and *empathy* (towards the respective patient).

### Quantitative results

All descriptive data are presented in Table 1.

**Table 1:** Mean and standard deviations of all dependent variables

Dependent Variables	VR			Conv.			
	Total *M (SD)*	Speaker *M (SD)*	Observer *M (SD)*	Total *M (SD)*	Speaker *M (SD)*	Observer *M (SD)*	scale
SLS-OLE							
Perceived Learning	2.50 (0.97)	2.49 (1.02)	2.51 (0.97)	4.50 (0.88)	4.68 (0.89)	4.40 (0.87)	1–6
IMI							
Interest/Enjoyment	3.87 (1.30)	3.82 (1.23)	3.90 (1.36)	5.33 (1.21)	5.51 (1.26)	5.21 (1.18)	1–7
BS							
Behavior	4.03 (1.41)	4.19 (1.42)	3.94 (1.43)	5.80 (0.94)	5.82 (1.11)	5.79 (0.84)	1–7
Emotion	4.69 (1.46)	5.19 (1.14)	4.39 (1.57)	6.06 (0.91)	5.99 (0.90)	6.11 (0.93)	1–7
Overall	3.13 (1.38)	3.47 (1.42)	2.92 (1.34)	5.73 (1.14)	5.67 (1.43)	5.76 (0.94)	1–7
SES							
Affective	2.57 (0.81)	2.84 (0.68)	2.41 (0.85)	3.48 (0.73)	3.69 (0.76)	3.34 (0.69)	1–5
Cognitive	3.51 (0.99)	3.82 (0.71)	3.31 (1.12)	4.23 (0.67)	4.37 (0.63)	4.14 (0.69)	1–5

#### Estimated learning success (H1)

**Fig. 3:**
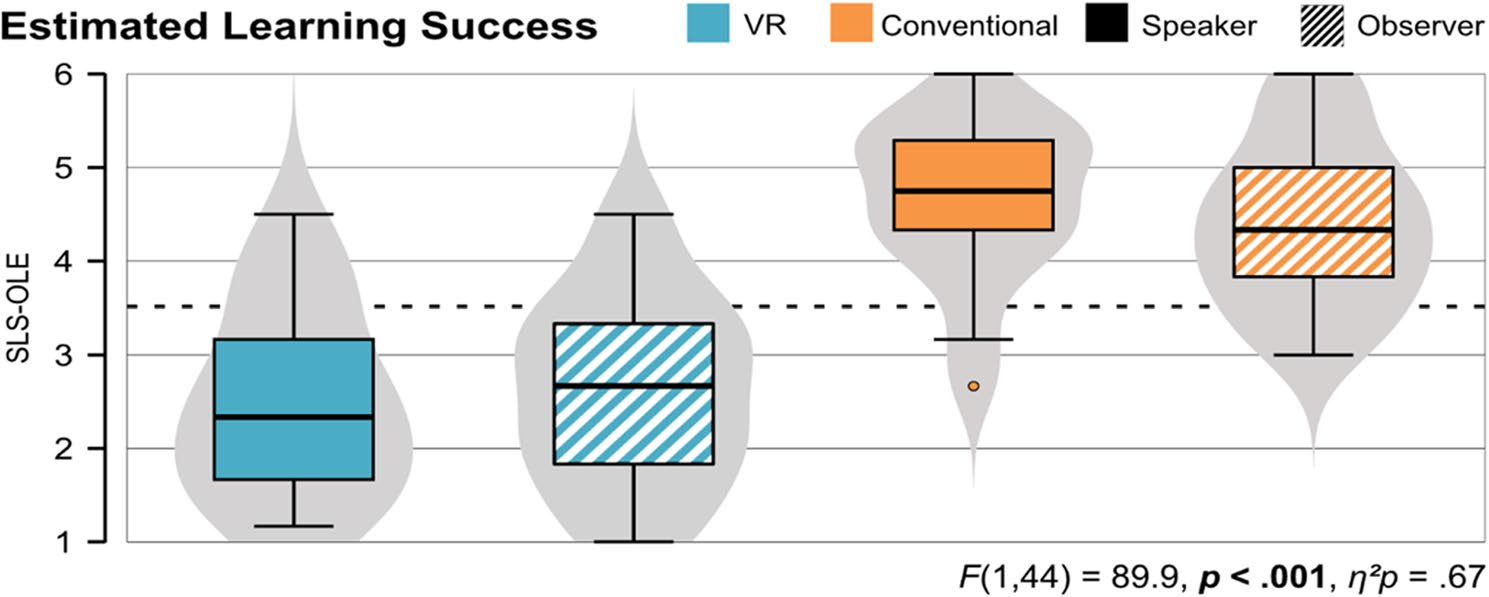
Comparison of the estimated learning success (SLS-OLE) between speakers and observers for both learning units

Regarding the estimated learning success (Fig. 3), the learning units (VR and conventional) significantly differ, *F* (1, 44) = 89.9, *p* < .001, *η*^*2*^*p* = 0.67. There was no interaction effect (*F* (1, 44) = 0.52, *p* = .474, *η*^*2*^*p* = 0.01), as well as no significant difference between speakers and observers (*F* (1, 44) = 0.51, *p* = .480, *η*^*2*^*p* = 0.01). The conventional unit condition was rated higher on average than the VR unit condition for all participants (*speaker* and *observer*).

After each conversation, the participants had to make a diagnosis. Table 2 shows that the respective disorders were recognized well as 86% in the conventional and 80% in the VR units made a correct diagnosis, regardless of the patient.

**Table 2:** Amount of correct or wrong diagnosis of the three patient personas for both learning units

Patient	VR				Conv.			
	*N*	Right	Wrong	No data	*N*	Right	Wrong	No data
Caro	19	18 (94%)	1 (5%)	-	15	15 (100%)	-	-
Emily	16	12 (75%)	4 (25%)	-	19	12 (63%)	4 (21%)	3 (15%)
Hannes	15	10 (66%)	3 (20%)	2 (13%)	16	16 (100%)	-	-
Overall	50	40 (80%)	8 (16%)	2 (4%)	50	43 (86%)	4 (8%)	3 (6%)

#### Motivation (H1)

**Fig. 4:**
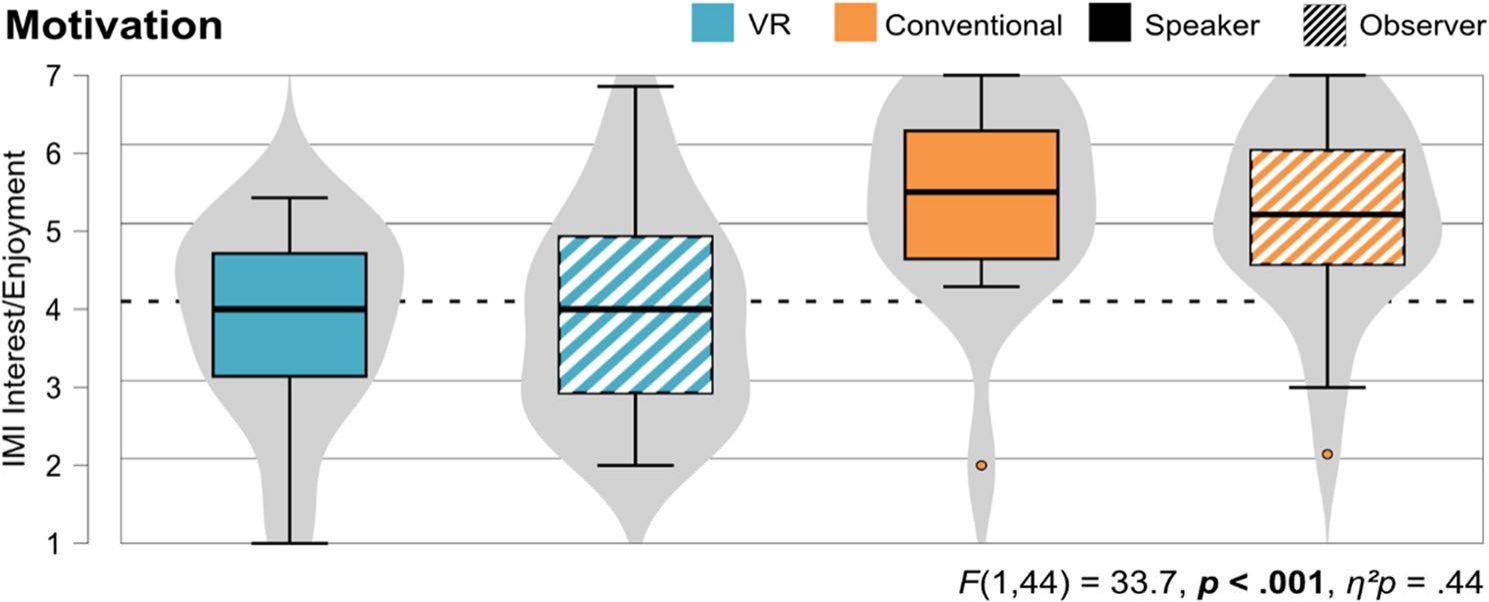
Comparison of the motivation (IMI) when using the learning units between speaker and observer for both learning units

Regarding the motivation during the learning units, ratings significantly differ between the two units (*F* (1, 43) = 33.7, *p* < .001, *η*^*2*^*p* = 0.44), but there was no interaction effect (*F* (1, 43) = 0.52, *p* = .475, *η*^*2*^*p* = 0.01), nor a difference between speaker and observer (*F* (1, 43) = 0.15, *p* = .702, *η*^*2*^*p* = 0.00). Figure 4 shows the violin plots for speaker and observer for each learning unit, showing a higher motivation during the conventional unit, than the VR unit.

#### Believability (H2)

**Fig. 5:**
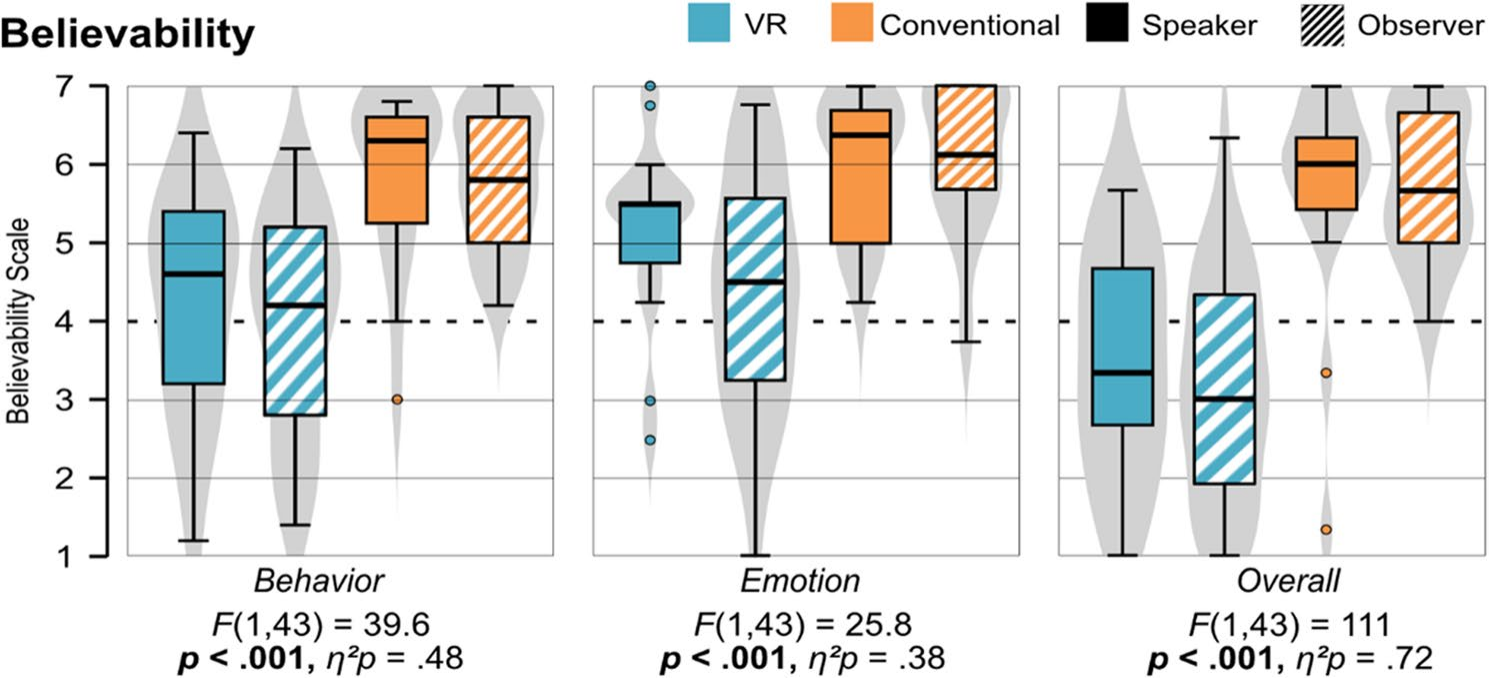
Comparison of the believability of the two patient types between speakers and observers for both learning units

Regarding the believability, the conventional and VR units significantly differ between all three subscales: behavior (*F* (1, 43) = 39.6, *p* < .001, *η*^*2*^*p* = 0.48), emotion (*F* (1, 43) = 25.8, *p* < .001, *η*^*2*^*p* = 0.38), and overall (*F* (1, 43) = 111, *p* < .001, *η*^*2*^*p* = 0.72), while there were no significant interaction effects or differences between speaker and observer. Figure 5 shows that the conventional unit was rated higher in all three subscales compared to the VR unit. Nonetheless, the VR unit was rated over average regarding behavior and emotion, but under average regarding the overall believability.

#### Empathy (H2)

**Fig. 6:**
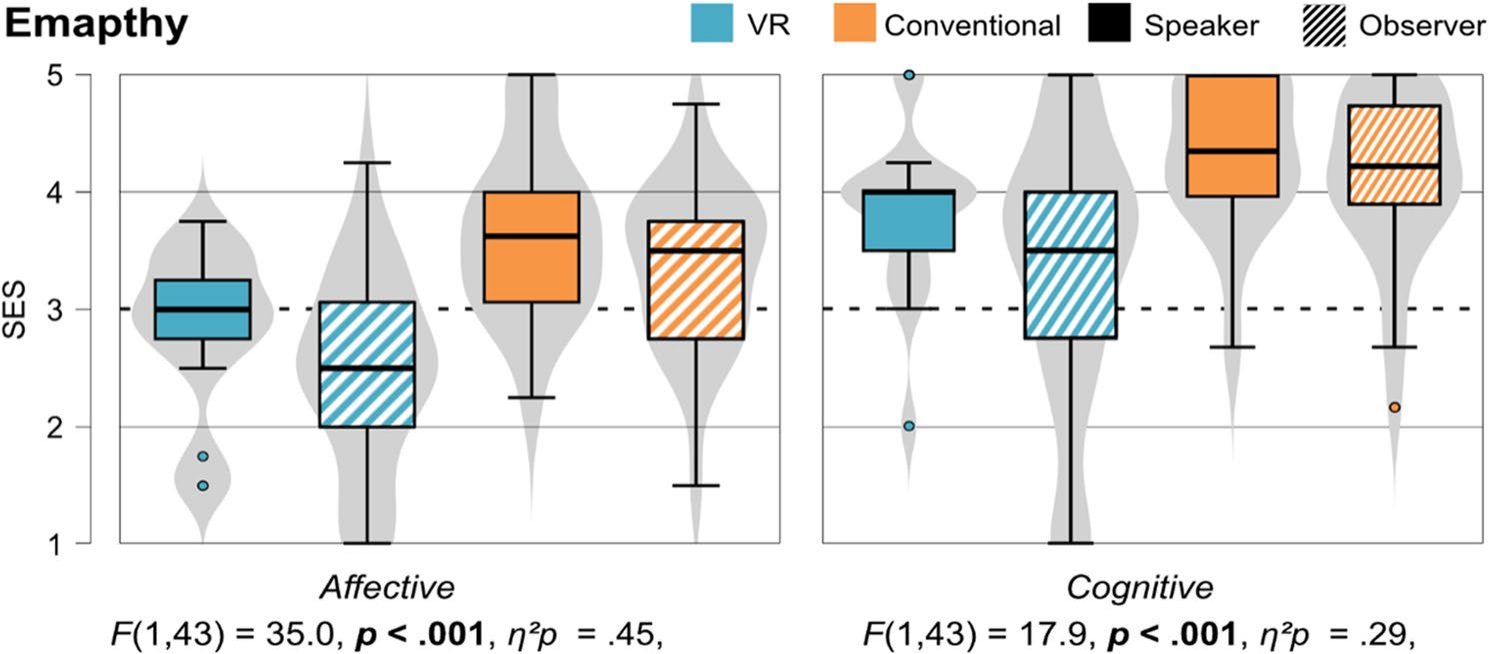
Comparison of the empathy (SES) towards the two patients between speakers and observers for both learning units

The affective subscale measuring empathy towards the respective patients (virtual vs. standardized) differs significantly, *F* (1, 43) = 34.96, *p* < .001, *η*^*2*^*p* = 0.45. There was also a significant difference between speaker and observer (*F* (1, 43) = 4.80, *p* = .034, *η*^*2*^*p* = 0.10), but no interaction effect (*F* (1, 43) = 0.08, *p* = .781, *η*^*2*^*p* = 0.00), indicating that the difference between the learning units is independent from which role the students had (speaker or observer). Regarding the cognitive subscale, we also found a significant difference between the learning units (*F* (1, 43) = 17.9, *p* < .001, *η*^*2*^*p* = 0.29), but no difference between speaker and observer (*F* (1, 43) = 0.78, *p* = .383, *η*^*2*^*p* = 0.02), nor an interaction effect (*F* (1, 43) = 3.39, *p* = .073, *η*^*2*^*p* = 0.07). Figure 6 shows the violin plots for both subscales for speakers and observers for each learning unit.

### Qualitative results

For the analysis of the qualitative results, we looked at the interviews we conducted in the end of the two runs comparing both learning units. In total, 43 students participated in the interview, while 17 of them were speaker and 26 observer. First, participants were asked to choose their preferred unit and then to explain their decision. Results yielded in the majority of the students (81.4%) preferring the conventional unit with the standardized patient, confirming the quantitative results. 6.98% chose the VR unit with the virtual patient, 9.30% were indecisive, and 2.33% gave no information.

#### Reasons for preferring the standardized patient

The reasons why the students preferred the conventional unit with the standardized patient over the VR unit were the following, presented in descending order by number of mentions: *insufficient speech recognition* (56), *lack of interaction* (33), *no real human* (15), and *inexplicable reasons* (14).

Therefore, the majority of reasons students gave for disliking the VR unit, as well as for preferring the conventional unit, were due to *insufficient speech recognition*, namely the virtual patient answering with phrases like ”*I do not understand you*,* can you repeat that*”. The students described that the insufficient speech recognition meant that they simply had to rattle off the questions or read them out exactly instead of pre-fumbling them themselves (20) as well as they were unable to ask their own questions or formulate them freely (10). The virtual patient’s repeated expressions of misunderstanding led to student frustration, which hindered enjoyment (5) and prevented the development of a natural conversational flow (4). Furthermore, students reported that this also made it difficult for them to engage with the patient (2), they were uncertain whether the responses were intentionally scripted or the result of technical malfunctions (1), and did not consider such repetition useful for learning success (1). However, in the context of the incorrect speech recognition, five students also mentioned that the VR application was not yet fully developed and that if speech recognition worked better, they would otherwise rate the VR unit more highly.

In addition, students mentioned a *lack of interaction* during the conversation with the virtual patient. While most of them just described the conversation with the standardized patient as ”*more interactive*” and ”*not just mere question-and-answer sessions*” in which ”*the patient can also react to what is said*”. The most frequently named reasons for the higher level of interaction in the conventional unit was the fact that it was not possible to respond empathetically to responses in the VR unit or to use reflective techniques (e.g., mirroring the patient’s emotions) (17):

*”[In the conventional unit] there was much more empathy and the ability to say: ’I understand how you feel’ Here it was really just a symptom query and no interaction”.*

The virtual patient, therefore, failed to respond appropriately which made it difficult to establish a natural interaction. In addition, students named that the non-verbal behavior of the standardized patients made the conversation more interactive as it seemed that the standardized patient reacted to the students questions with their body language (5). Lastly, they mentioned the spontaneity and dynamism of the responses of the standardized patient versus the virtual patient as an aspect that made the conventional unit more interactive (4). Even though participants could theoretically freely ask questions, the virtual patient only had pre-formulated answers to diagnostic questions. Another reason, why students preferred the conventional unit was the fact, that the virtual patient is *no real human*. While a total of 15 students stated that the VR unit performed poorly because it was “*not a real person*”, only a few specified this reason further. Two students said that the virtual environment was too artificial or that the comic style of the virtual patient was disturbing, one described the conversation with the standardized patient as a “*more natural conversation atmosphere*” and another said that the learning success seemed higher when you have to think about what to say to the real person in front of you. The last aspect can be summarized as *inexplicable reasons* and includes statements such as:

*“I found the standardized patient unit somehow just better”.*

It seems that some participants found it difficult to justify their choice or to put it into words. Nevertheless, it cannot be excluded that the insufficient speech recognition is not the main problem here too.

#### Advantages and disadvantages of both learning units

Even though the insufficient speech recognition was one of the most common reasons for the preference for the conventional unit, there were still comments in favor of VR, as well as criticism regarding the standardized patient. For example, students expressed advantages of VR if speech recognition worked better, or disadvantages of the conventional unit, which they could imagine better in VR. Those are listed in Table 3. In VR, the tablet as an aid for the questions that can be asked in the interview seemed to be, particularly, positively evaluated (9), as well as the detailed answers that the virtual patients were able to give (5). In addition, the students found VR to be a kind of easy introduction (4), which can be used, for example, before acting with a standardized patient, which is standardized for everyone (3), and, thus, offers a good opportunity to practice (2). Furthermore, they described it as:

*”It’s also better to “cheat”*,* because somehow it’s not as unpleasant as sitting opposite an acting patient or a real patient and always having to look at my list”.*

Nevertheless, some students also found VR distracting (6), too artificial (2), and found it difficult to interpret the virtual patient’s body language (2) and pauses in speech (2). The main advantages of the conventional unit were the non-verbal communication (4) and the feeling of being able to build a relationship with the patient through natural interaction (4). The age (3) and limited or different skills of the actors (1) were mentioned as negative factors.

**Table 3:** Advantages and disadvantages of both learning units independent from previously described reasons

Unit	Advantages	Disadvantages
VR	• Tablet as a tool (9) • Detailed responses from the virtual patient (5) • Cool/fun (4) • Easy introduction before working with standardized patients (4) • Standardization (3) • Low threshold for use (3) • More possibilities for expression, e.g., crying (3) • Good for practice (2) • Age of the patients (1) • Availability (1)	• VR, especially the operation of the tablet, is too distracting (6) • Artificial/comic-like presentation (2) • Difficult to empathize and interpret body language (2) • Response options unclear (e.g., pauses in speech) (2) • Humanity missing (1) • Not high-resolution enough (1) • VR headset tiring to wear (1)
Conventional	• Non-verbal communication (4) • Pleasant & natural interaction that builds rapport (4) • Improvisation skills (1) • “Holding out” when there is no response (1)	• Age of actors (3) • Skills limited (e.g. ability to cry) (1) • Actors all different (1)

#### Believability of both learning units

**Fig. 7:**
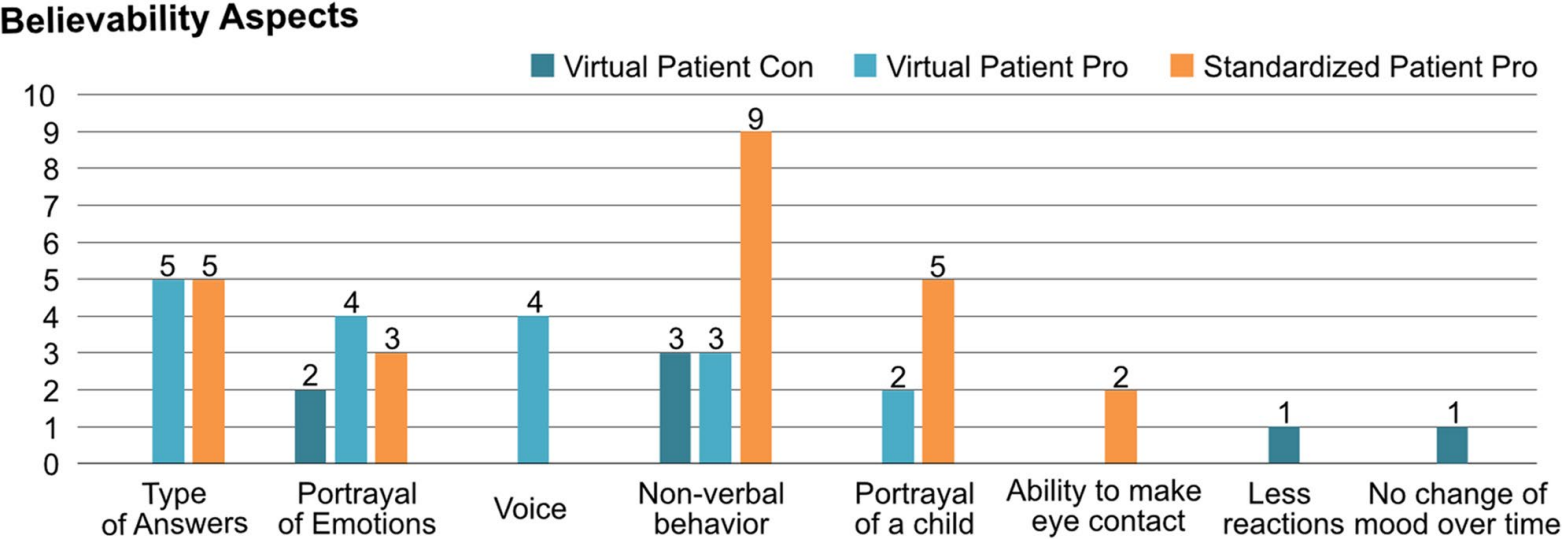
Amount of aspects pro and contra believability for both patients

Lastly, we collected statements about aspects that made the respective patient appear believable or not. Figure 7 shows the comparison of both units. 18 statements emphasized the believability of the virtual patient: The type of answers (5), the portrayal of different emotions (4), the voice and tone of voice (4), non-verbal behavior and posture (3), as well as the portrayal of a child (2) were mentioned. However, seven statements also emphasized aspects that made the virtual patients appear less believable: Non-verbal behavior (3), few emotional expressions (2), few reactions (1), and no change in mood over the course of the conversation (1). For the standardized patient, we collected 24 statements emphasizing the believability of the actor: The non-verbal behavior (9), the type of answers (5), the portrayal of a child (5), the portrayal of different emotions (3), and the ability to make eye contact (2). We also collected nine statements, that emphasize a similar behavior of the students towards both patients, indicating a general believability of the virtual patient in the direct comparison with the standardized patient. Similar aspects of both patients were the believable portrayal of emotions (4), and the attitude with which the students entered the interview (2). In addition, one student said that they would have been overwhelmed if the patients had cried in both units (virtual or standardized patient) and another said that the virtual patient would have been similarly believable if the speech recognition had worked better.

### Objective results

#### Error analysis of our speech model

The analysis of the interaction logs (Table 4) resulted in a total amount of (42.7%) correct allocated answers of the virtual patients, and 57.3% errors by a total amount of 1037 answers to the participants questions. According to the procedure of Graf et al. [4], we distinguish between *concept errors*, which are errors that appear when there is no predefined answer to a question in our concept, as well as *system errors*, which are due to a wrong allocation of wit.ai, the system we used for the speech recognition. While only 24.6% were *concept errors*, 75% were *system errors*. Moreover, we specify system errors into four different types of errors to further understand the issues of appearing. Thereby, Table 5 shows that most errors (37.1%) occurred because the system misinterpreted short utterances such as ”*Ehm*” or ”*ok*” – intended merely as acknowledgments – as new questions addressed to the virtual patient. Followed by errors (24.2%) that occur because the wording of the question deviated too much from all pre-formulated alternative questions. Another reason for errors (16.3%) was that the system interpreted the input too early, for example, when questions consists of very long sentences or when two questions were asked in one (i.e., ”*Why are you here today and what can you tell me about yourself in general?* ”). Lastly, 12.5% of the errors occur due to participants talking to the experimenter and so the system recognized it as a question that could not be allocated, or 10% were not recognized for no explainable reason.

**Table 4:** Amount of right and wrong allocations for 14 participants, tracked by the interaction logs. * Not-existing intention in our concept, ** wrong intention allocation by the system

	Asked questions in total	Correct allocation	Totalerrors	Concept* errors	System** errors
Amount	1,037	443	594	146	448
%		42.7%	57.3%	24.6%	75%

**Table 5:** Amount and definition of specified system errors

	System error a.	System error b.	System error c.	System error d.	System error e.
	Question not understood, although it is in the set of questions.	Alternative question does not exist.	System interpreted the voice input too early.	Short statements have already been recognized as input.	Participant spoke to experimenter or themselves.
Amount	45	108	73	166	56
%	10%	24.1%	16.3%	37.1%	12.5%

## Discussion

Our goal was to evaluate a VR unit using virtual patients with a conventional unit using standardized patients to compare advantages and disadvantages of both units regarding learning success, motivation, believability, and empathy in an early development process.

Standardized patients received higher ratings across all dependent variables. The results contradict other studies, which either showed that VR was equally effective compared to standardized patients [23], or even more effective [3, 9]. It should be noted here that the studies were either conducted in disciplines other than child and adolescent psychiatry (neurological examinations [3], ophthalmology [23]), dealt with clinical competencies other than communication skills (neurologic physical exam [3], clinical reasoning skills [23]), or used virtual patient systems that did not employ VR via an HMD, but used a computer-based case study displayed on a desktop screen [23].

In our study, however, the potential of virtual patients appears to have been limited primarily due to inadequate speech recognition, which hindered effective interaction. These results confirm findings from similar studies, which also found negative ratings of the patient system due to malfunctioning speech recognition, as users disliked the repetition of the conversation and, therefore, might rate it less positively overall [15]. Speech recognition limitations observed in the VR condition should be considered implementation constraints of the current system rather than evidence against the validity of VR-based therapeutic or learning interactions. Nevertheless, participants identified several advantages of the VR unit. They appreciated its clear structure, consistency, the low barrier to entry, particularly, for initial practice, and its suitability as a preparatory tool before engaging with standardized patients. Furthermore, the expressive potential and youthful appearance of the virtual patient was perceived positively, but the virtual patients can also feel artificial and less emotionally engaging. Some participants reported feeling more at ease interacting with a real human being—at times for reasons they could not clearly articulate. Standardized patients were particularly valued for their ability to convey nonverbal behavior, respond spontaneously, and engage in dynamic interpersonal exchanges. Many participants felt they could form a more authentic connection with a real person, which allowed for more genuine emotional expression during the interaction. Nevertheless, standardized patients also have limitations in standardization, emotional expression, and actor variability.

### Self-estimated learning success & motivation of both learning units (H1)

While in terms of self-estimated learning success, the conventional learning unit was rated significantly better than the VR unit (for both speakers and observers) diagnostic accuracy did not differ between conditions. This discrepancy indicates that subjective learning evaluations may not align with objective performance outcomes. Hence, despite its limitations, the virtual patient was capable of conveying relevant information during the interaction to allow students make the right diagnosis. The objective error analysis provides an additional perspective on diagnostic performance. It helps to link observable diagnostic mistakes to educational outcomes, as fewer or less severe errors indicate better acquisition and application of clinical reasoning skills. Moreover, it allows us to differentiate between errors that are conceptual (concept errors) and those that are technical (system errors), which can inform future improvements of the VR system and its integration into the learning process. The fact that system errors predominated underscores that the usability and interaction mechanics of the VR system are integral to its educational value, as they directly shape learners’ ability to engage with the virtual patient.

The qualitative data provided reasons for the poor assessment of learning success of the VR learning unit. Insufficient speech recognition in particular seems to have disturbed the flow of conversation, which is why students were less able to concentrate on the content. Students were mainly concerned with formulating sentences correctly so that the system would understand them, which could have limited their ability to pay attention to the virtual patient’s answers, which in turn led retrospectively to a lower rating in terms of the assessed learning success. Additionally, operating the VR system itself distracted some participants, which represents an additional component that did not have to be considered in the conventional unit. However, a learning effect in VR might eliminate this disadvantage for future uses. Despite the poor evaluation of VR, there were also positive aspects that could contribute to a potential learning success. For example, students described the unit as an easy introduction to the interview situation or as a good preliminary stage for practicing before meeting the acting patient without any worry, confirming findings from Sapkaroski et al. [9], that highlight the potential of virtual patient systems for individual learning without the pressure of evaluation by others. Some studies described how the virtual patient’s carefully formulated answers ensured a high level of standardization of the learning content and also addressed topics that the students would not otherwise have thought to address or ask. In addition, although some described the tablet as distracting, it was perceived by others as a support to learn the structure of the interview, even though a real tablet with the same content was also provided in the acting condition. It seems that the students were less uncomfortable looking up something on the tablet from time to time with the virtual patient than they were with the actor in the conventional session.

The qualitative data also provided reasons for the preference of the conventional learning unit. Students emphasized that the nonverbal behavior was more pronounced than in the VR condition. In addition, the interaction with a real person created a kind of patient-physician relationship. Some also preferred the standardized patient simply because the encounter involved a real person, which prompted them to pause and choose their words more carefully. Furthermore, it seemed difficult to explain why a real person is better or what was missing in the virtual patient.

In terms of motivation during the learning unit, the conventional unit also performed significantly better than the VR unit, although the values for the VR unit were average. Students described the VR unit as cool and fun, but also said that they would have rated it better if the speech recognition had worked better.

### Believability & empathy transfer (H2)

When asked about the believability of the respective patients, meaning how convincingly the actor was able to play the patient and the virtual character was able to portray the patient, as well as the question of how empathetic the respective patients felt towards the actor, again the standardized patients received higher values than the virtual patient. Nevertheless, the virtual patient’s behavior and emotions were still rated believable, confirmed by the qualitative results. For example, the expressed emotions by the virtual patients’ body language and voice tone, as well as the accuracy of its’ answers were received as believable. Some participants stated that they liked the more youthful appearance compared to the standardized patients. There was some disagreement among the students regarding believability, with some citing the non-verbal behavior of the virtual patients as believable, while others considered this to be the one thing that made them not believable enough. Also, the portrayal of a child patient seems to be not as important as expected, as only a few mentioned it as a believable aspect of the virtual patient, but more emphasized that the adult actors believably portrayed child patients. The low response variance and the fact that no changes in emotions were shown over the course of the interview also made the virtual patient unbelievable.

Regarding empathy, the qualitative results revealed that students felt more easily able to interpret the emotional behavior of the actors and, therefore, could build rapport with them. Nonetheless, the descriptive data from the quantitative results show that virtual patients are also rated positively in terms of the cognitive empathy subscale, albeit significantly less positively than the standardized patients. This could indicate that cognitive empathy, i.e., understanding the patient’s situation, was also felt in VR. This is contradict with findings from related work, showing that negative aspects were the difficulty of empathizing with the virtual patient [15]. Hence, our results point out more into the direction of Sapkaroski et al. [9], who, in their comparison of VR and role-playing, even found a significantly better development of empathic communication in the VR condition. They argue that VR’s high level of immersion allows users to “immerse” themselves more deeply in the patient’s situation, which in turn increases their receptivity to empathic behavior. In our case, the lack of interaction (one reason for the preference of the conventional unit) appears to have prevented students from expressing empathy towards the virtual patients, as they could not convey any feelings or emotions, but only trigger questions.

### Potential of virtual patients for child and adolescent

Psychiatry Although virtual patients have been extensively studied and applied in general medical education [31], their use in psychiatric education remains a relatively new development. A systematic review and meta-analysis provided evidence that standardized-based psychiatry education effectively enhances the knowledge, clinical skills, and attitudes of medical students and healthcare professionals [32]. The authors of a recent meta-analysis [33], that identified 46 studies addressing the use of virtual patient interventions in undergraduate psychiatry education pointed out the underrepresentation of studies focusing on pediatric patients and young adults with mental illness.

In our comparison, subjective learning success and motivation for the VR application were rated below average compared to the standardized patient unit. This rating is probably mainly due to speech recognition, in line with the results of other studies [15]. Nonetheless, students liked the standardization, the clear structure and the low barrier of starting a conversation in VR, confirming results of the review of Milne et al. [15], that described virtual patient systems as ’risk-free learning environment’, as they were not actual patients. With regard to the dependent variables that specifically concern the virtual patient itself, namely believability and empathy, our results show values above average, albeit significantly worse than the standardized patient, which indicate agreement with the believable behavior of the virtual patient and the perceived empathy behavior towards it. The emotions of the virtual patients were recognized and found to be believable. The poor rating of overall believability can, therefore, probably also be attributed to the overall impression made by the poor speech recognition. In particular, the content of the virtual patients’ responses was also perceived positively, and the students arrived at the correct diagnosis comparatively well, despite the possibly poor course of the conversation. The use of the virtual patient system, particularly in the field of psychiatric diagnosis, therefore, seems to be justified and confirms the results of other studies in the field of adult psychiatry [5]. Although the fact that the actors were not children was described as disturbing by only a few, others cited this as an advantage of VR. In summary, the use of VR is also promising in the field of child and adolescent psychiatry, as virtual patients can: (a) believably represent the complexity of the thought structures of mentally ill children and adolescents, (b) enabling empathic understanding of the patient’ situation, (c) be diagnosed, and (d) provide a safe setting for practicing the structure of diagnostic interviews. Above all, improved speech recognition is required so that the necessary flow of conversation can be guaranteed.

### Limitation and the integration of AI in future work

The most significant limitation of our virtual patient system appears to be the insufficient speech recognition preventing the virtual patient from receiving a fair comparison to the standardized patient. The result can be described as the one problem that is most frequently documented in current virtual patient systems [15]. Our objective results align with students’ reports that no real interaction — for example, expressing empathy toward the patient — was possible in the conversations: Most errors appeared due to the system recognizing statements, such as “*ehm*” or “*ok*”, to agree with the virtual patient’s answer, already as a new question. The problems with the speech recognition thus led to a lack of conversational flow and interaction between the student and the virtual patient, resulting in a poor evaluation of the conversation in this unit, especially in direct comparison with the standardized patient. It is, therefore, not possible to determine exactly to what extent this problem influenced the overall evaluation of the virtual patient. In a next step, a revision of the speech recognition is, therefore, urgently needed, which could be achieved through the extended integration of AI [33, 34].

In the current state of our application, we only use the AI tool wit.ai to recognize the speech input and to assign a predefined intention to the students’ questions, which then plays a corresponding pre-recorded audio file, as also used in similar virtual patient studies [3, 31]. Further integration of generative AI models, such as OpenAI’s GPT, has not yet been carried out for the following reasons. Firstly, AI-generated responses are not entirely controllable [22]. Therefore, for example, Ayers et al. [22] concluded that chatbots such as ChatGPT could be used in the future to relieve doctors by formulating empathetic and high-quality responses, but only as a draft that would then have to be reviewed and, if necessary, adapted by medical staff. This human control is relevant because there is no guarantee of clinical accuracy, and ChatGPT can provide medical information that sounds plausible but is incorrect or incomplete (“hallucinations”). However, the study did not focus on teaching medical content, but rather on providing medical advice to patients, which requires even greater ethical assessment. Consequently, in our case of teaching psychiatric interviews, an AI-generated learning content could vary, or the AI could theoretically output content that, particularly in the field of psychiatric diagnosis, represents nuances that could point to one diagnosis or another. This raises the question of what the goal of the application should be: to convey accurately portrayed patients or to teach conversation skills, i.e., asking the important questions for making a diagnosis. However, it should be noted that the responses of the standardized patients cannot be entirely predetermined as well and that they may invent aspects that do not fit the given clinical picture either [35]. In the case of virtual patients, careful prompt engineering and, ideally, specialized training data for the AI model would be crucial for the accuracy of the virtual patients’ generated responses. Secondly, AI-generated responses would not be the same for all learners, which also makes comparability and standardization difficult. However, the same applies to standardized patients, as this also depends heavily on the ability of the actors [36]. Thirdly, even though AI-generated computer-generated voices are becoming increasingly better and more human-like [37], it is difficult to convey emotion in the voice [38].

For our application, a combination of pre-formulated as well as AI-generated answers from the virtual patient could be a possible solution. The conversation would mainly consist of pre-formulated answers that have been checked by a psychiatrist, and in the event of an unforeseen question for which no pre-formulated answer exists, an AI-generated answer would follow. This could improve the system’s ability to better recognize alternative question formulations, distinguish between questions and statements (such as “I understand you”), identify when a question is complete, and differentiate whether the user is addressing the virtual patient or, for example, another person is in the room.

Following this proposed revision through the expanded integration of AI systems such as the GPT models, we want to evaluate actual learning success. Objective criteria such as exam results after using the VR application or usage within the objective structured clinical examination (OSCE) could be considered for this purpose. It would also be interesting to explore the idea of using VR as a preparatory tool for learning units with the standardized patient. In a comparison of two learning groups, one group could prepare with the revised VR application, while another could prepare with conventional methods such as reading case studies. The subsequent behavior toward the standardized patient could then be compared with each other.

### Implications for the design of virtual patient systems

However, the results caused by insufficient speech recognition ensure that an explicit comparison is made, paying attention to aspects that might otherwise have been overlooked. For example, the virtual characters generally succeeded in accurately representing child patients and their emotions. This has implications for the design of future virtual patient systems. Since the combination of multimedia design elements (visual, auditory, and interactive) can be beneficial in terms of learning psychology, according to Sapkaroski et al. [9], the animation of the respective virtual patients’ body language must be improved. First, it is important to increase their believability [4], to let students interpret their non-verbal behavior, as well as to prevent misinterpretation of it. The latter means, for example, that pauses in speech by the virtual patient are designed in such a way that they are recognized as such, so that the user knows when they can ask questions again. Regardless of the linguistic component, the interaction could be supplemented by eye contact. The lack of non-verbal behavior – in addition to the flow of conversation being disrupted by poor speech recognition – may also have contributed to the students noticing, in particular, that the virtual patient is not a real person, making it difficult to establish a relationship with them.

## Conclusions

From the current point of view, the virtual patient cannot yet match the standardized patient in terms of self-estimated learning success, motivation, believability, nor empathy. At the same time, the findings indicate that VR offers distinct advantages, such as a closer resemblance to children and a lower inhibition threshold, may support its use as a complementary training tool. In particular, VR could serve as a preliminary exercise before using a standardized patient, as it can help to learn the structure before the emotional component is added to the conversation. Given the prototype nature of the VR system and the impact of technical limitations such as speech recognition, broader generalizations should be made cautiously. Nevertheless, an improvement in speech recognition (for example through the increased use of AI) could also have a significant influence on the students’ assessments, which is why further investigation of virtual patient systems seems worthwhile for future research.

## Supplementary Information

Supplementary Material 1.

## Data Availability

Anonymous data of the results of the evaluations could ask for the authors.

## Footnotes

1 ©Meta, https://wit.ai/, April/2025.

2
www.audiotranskription.de/en/f4-features/automatic-transcription/.

3
www.maxqda.com/.

## Abbreviations

AI: Artificial Intelligence
BS: Believability Scale
HCI: Human-Computer-Interaction
ICD-10: International Statistical Classification of Diseases and Related Health
Problems: 10th Revision
IMI: Intrinsic Motivation Inventory
LLM: Large Language Model
OSCE: Objective Structured Clinical Examination
SES: State Empathy Scale
SLS-OLE: Student Learning and Satisfaction in Online Learning Environment
VR: Virtual Reality
