# Supplementary material for: Virtual vs. standardized patients - a preliminary comparison of advantages and disadvantages of a VR and a conventional learning unit for medical students in child and adolescent psychiatry
Source: BMC Med Educ. 2026 Feb 17;26:339. doi: 10.1186/s12909-026-08760-4 (PMC12930773; doi:10.1186/s12909-026-08760-4)
Supplement: Supplementary file 1 — Supplementary Material 1. [file 12909_2026_8760_MOESM1_ESM.pdf]

Virtual vs. Standardized Patients - A Preliminary Comparison of Advantages and Disadvantages of a VR and a Conventional Learning Unit for Medical Students in Child and Adolescent Psychiatry

- Supplementary material -

Linda Graf, Vera Golz, Sophia Prehn, Maic Masuch, and Gertraud Gradl-Dietsch

Intervention study – Instruments

Please enter your 4-digit code:

Which round are you in?

1. Round	2. Round

Which learning unit have you just completed?

Standardized patient unit	VR unit

Did you just see a male [actor/virtual character] or a female [actor/virtual character]

female [actor/virtual character]	male [actor/virtual character]

Did you conduct the conversation, or did you observe it?

I did conduct the conversation.	I did observe the conversation.

What diagnosis would you make for the patient?

<u>Student Learning and Satisfaction in Online Learning Environments Instrument (SLS-OLE) – DiLoreto et al. 2022</u>

Perceived Learning	Strongly Disagree	Mostly Disagree	Slightly Agree	Moderately Agree	Mostly Agree	Strongly Agree
I am pleased with what I learned in the [standardized patient/VR] unit.						
The [standardized patient/VR] unit enhanced my understanding of the content.						
I learned less in the [standardized patient/VR] unit than I anticipated.						
I learned skills that will help me in the future.						
The learning activities promoted the achievement of student						
The [standardized patient/VR] unit contributed to my professional development.						

Believability Scale (BS) – Guo et al., 2023

Behavior	Strongly Disagree	Disagree	Somewhat Disagree	Neither Agree nor Disagree	Somewhat Agree	Agree	Strongly Agree
The [actor's/virtual patient's] behavior drew my attention.							
I felt the [actor's/virtual patient's] behavior was coherent and natural.							
I think the [actor's/virtual patient's] behavior was easy to understand.							
I felt the [actor's/virtual patient's] behavior was appropriate to the context.							
I felt sometimes the [actor/virtual patient] behaved inappropriately.							

Emotion	Strongly Disagree	Disagree	Somewhat Disagree	Neither Agree nor Disagree	Somewhat Agree	Agree	Strongly Agree
I felt that the [actor/virtual patient] was capable of having feelings.							
I felt that the [actor/virtual patient] expressed emotions.							
I felt that the [actor's/virtual patient's] expressed emotions were easy to understand.							
I felt that the [actor's/virtual patient's] expressed emotions were appropriate to the context.							

Overall Believability	Strongly Disagree	Disagree	Somewhat Disagree	Neither Agree nor Disagree	Somewhat Agree	Agree	Strongly Agree
I felt that the [actor/virtual patient] was believable.							
I felt that the [actor/virtual patient] behaved like a real person.							
I enjoy the interaction with the [actor/virtual patient]							

State Empathy Scale (SES) – Shen 2010

Affective Empathy	Strongly Disagree	Disagree	Neither Agree nor Disagree	Agree	Strongly Agree
The [actor's/virtual patient's] emotions are genuine.					
I experienced the same emotions as the [actor/virtual patient] when watching the [actor/virtual patient].					
I was in a similar emotional state as the [actor/virtual patient].					
I can feel the [actor's/virtual patient's] emotions.					

Cognitive Empathy

I can see the [actor's/virtual patient's] point of view.					
I recognize the [actor's/virtual patient's] situation.					

I can understand what the [actor/virtual patient] was going through in the situation.					
The [actor's/virtual patient's] reactions to the situation are understandable.					

Intrinsic Motivation Inventory (IMI) – Deci & Ryan 2020

Interest/Enjoyment	Strongly Disagree	Disagree	Somewhat Disagree	Neither Agree nor Disagree	Somewhat Agree	Agree	Strongly Agree
I enjoyed the [standardized patient/VR] unit very much.							
The [standardized patient/VR] unit was fun to do.							
I thought the [standardized patient/VR] unit was a boring activity.							
The [standardized patient/VR] unit did not hold my attention at all.							
I would describe the [standardized patient/VR] unit as very interesting.							
I thought the [standardized patient/VR] unit was quite enjoyable.							
While I was doing the [standardized patient/VR] unit, I was thinking about how much I enjoyed it.							

Interview Questions

1. Which learning unit (VR/conventional) did you like better and why?

2. How believable did the virtual character/actor portray the respective patient?

    a. What did you particularly like?
    b. What was missing?
